# Targeting Adults for Supplementary Immunization Activities of Measles Control in Central China: A Mathematical Modelling Study

**DOI:** 10.1038/s41598-018-34461-0

**Published:** 2018-10-31

**Authors:** Ka Chun Chong, Chi Zhang, Katherine Min Jia, Benny Chung Ying Zee, Tongyong Luo, Lei Wang, Greta Chun Huen Tam, Riyang Sun, Maggie Haitian Wang, Xuhua Guan

**Affiliations:** 1 JC School of Public Health and Primary Care, The Chinese University of Hong Kong, Hong Kong, China; 2 Clinical Trials and Biostatistics Laboratory, Shenzhen Research Institute, The Chinese University of Hong Kong, Hong Kong, China; 3 Hubei Provincial Center for Disease Control and Prevention, Hubei, China

## Abstract

Routine immunizations and supplementary immunization activities (SIAs) have significantly improved measles control over the past two decades in China. Progress towards eliminating measles currently faces multiple challenges as the infection age increases, and adult-targeted SIA strategies are being considered. This study developed an age-stratified susceptible-exposed-infectious-recovered model using a recently published contact matrix to depict measles transmissions between individuals in seven age groups. Hubei, a high measles-incidence province in central China, was the selected setting. The baseline scenario was calibrated by fitting with the 2012–2015 age-stratified incidence data. SIAs targeting multiple age groups were simulated. Adult-targeted (>29 years) two-year SIA cycles produced the greatest annual incidence rate decrease, reducing incidences by half over a long timespan with 90% coverage levels. Incidences could remain below 10/100,000 until 2030 if SIAs were provided to individuals ≥6 years old with at least 50% coverage. These findings will help officials prioritize supplementary vaccination strategies. Public health officials in China should consider adult-to-adult transmissions and provide adult-targeted SIAs. Although officials have reported approximately 90% SIA coverage in the past, SIAs for the adult population should be provided on shorter intervals, particularly for the aging population with decreased immunity.

## Introduction

Measles, a highly contagious infectious disease caused by the measles virus in the paramyxovirus family, creates a high burden of childhood mortality and morbidity globally. Before the measles vaccine was licensed in 1963, measles was globally widespread: over 90% of children were infected once before they turned fifteen, resulting in >2 million deaths annually^1^. During the late-20^th^ century after the vaccine was introduced, the morbidity and mortality rates of measles were reduced by 74% and 85%, respectively, as compared to the pre-vaccine period^2^. After the Global Immunization Vision and Strategy was established in 2000, measles mortalities dropped from an estimated 550,100 in 2000 to 89,780 in 2016^3^.

Measles remain endemic in China, the largest country (population 1.3 billion) in the Western Pacific Region of the World Health Organization^4^, with the measles virus circulating across the country and measles incidences reported in all provinces in 2014^5^.The current measles control and prevention strategies are: i. immunization, including a routine immunization with the first dose of measles-containing vaccine (MCV1) given at 8 months of age, the second (MCV2) between 18–24 months of age, and province-level SIAs (e.g., mass immunization campaigns targeting all individuals in specific age groups regardless of their vaccination history); ii. measles surveillance with the national case-based system; and iii. infection control in healthcare settings and during outbreaks^4,6^. Under the 2006–2012 National Action Plan for Measles Elimination, the country increased routine immunization coverage rate, initiated SIAs to increase immunity levels among children of specific ages (e.g. 8 months–5 years) in a short period of time, and adopted emergency immunizations for outbreak control^7^. As a result of the nation-wide SIAs in 2010, measles incidences dropped to a record low of 0.46 cases per 100,000 people in 2012; however, this was followed by a resurgence of incidences in 2013 and 2014 (2.04 and 3.88 cases per 100,000, respectively)^4,5^, especially among unvaccinated young children nationally and adults over 15 years of age in certain localities^8^. To date, most areas remain in an endemic state, with accumulating susceptible hosts and seasonal outbreaks experienced both regionally and nationally^4,9^.

As the number of adult infections in China has increased, the aptitude of the current routine immunization strategy and supplemental SIAs for controlling future measles epidemics is in question. This study used mathematical model simulation to evaluate whether future SIAs should target older groups of individuals in China.

## Methods

### Setting and data collection

The Hubei province, located in central China and hosting 58 million residents, had a moderate-to-high disease incidence relative to other Chinese provinces with six prefecture-level cities identified as high-risk for measles outbreaks in 2015^10^. Like other provinces in China, Hubei had a low birth rate (approximately 10 per thousand people). The reported annual vaccination coverage for the first and second MCV doses in 2012 were approximately 95%. In addition to routine immunization, the Hubei Provincial Center for Disease Control and Prevention (Hubei CDC) conducted two SIAs, in 2009 and 2010. The 2009 SIA was administered between September and November, targeting children between 8 months–14 years old, whereas the 2010 SIA was administered between September 2010 and March 2011 and targeted age-eligible children who had not yet been vaccinated through routine immunization. Both SIAs were eventually reported with 98% coverage rates. The age distribution of measles cases in this province paralleled the national trend, with a persistent proportion of cases affecting adolescents and adults (>15 years). This demographic accounted for 21% of overall cases in 2006–12. On the other side of the spectrum, infant infections (age <8 months) increased from 16% in 2006 to 34% of all cases in 2012^11^.

**Figure 1:**
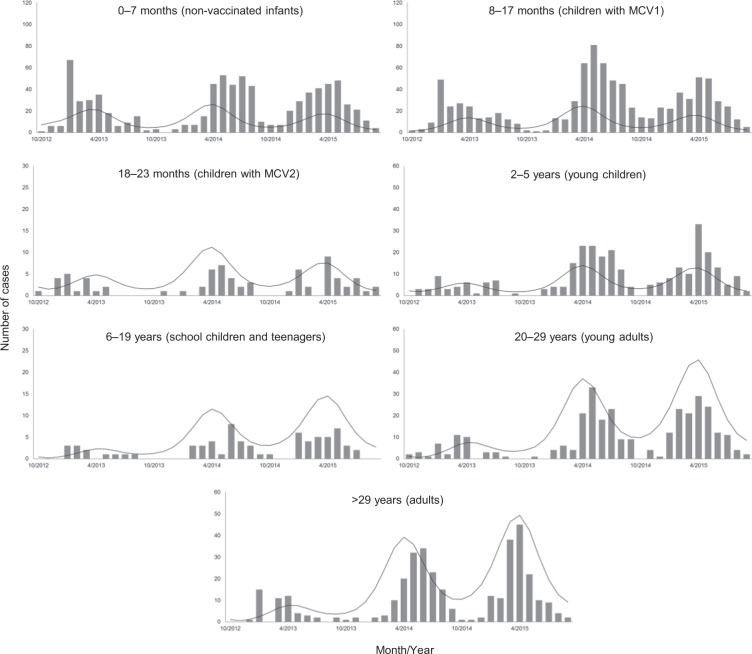
Observed (bars) and fitted (solid lines) measles cases stratified by age groups, 2012–2015.

This study used the notified number of measles cases in the Electronic Surveillance System of Hubei CDC to calibrate the baseline scenario. This surveillance system centralizes information regarding notifiable infectious diseases from all 17 counties in the Hubei province. Measles cases were defined based on the World Health Organization clinical and epidemiological standard^12^. Data were stratified into different age groups on monthly scale, including three epidemic periods from 10/2012–9/2015 (Fig. 1). Demographic information was collected from the Bureau of Statistics of Hubei^13^.

### Disease transmission model

**Figure 2:**
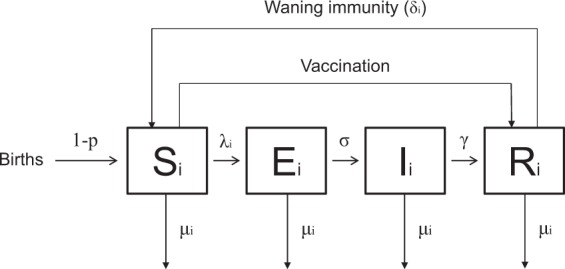
Age-stratified Susceptible-Exposed-Infectious-Recovered model for measles transmissions.

An age-structured compartmental model, common method to evaluate different infectious disease control strategies was extended to study the transmission dynamics and impacts of SIA interventions. Figure 2 depicts its essential features. The entire population was stratified into seven age groups (*i* = 1, 2, 3, …, 7): 0–7 months (non-vaccinated infants), 8–17 months (children with MCV1), 18–23 months (children with MCV2), 2–5 years (young children), 6–19 years (school children and teenagers), 20–29 years (young adults), and >29 years (adults). Susceptible individuals in the *i*-th age group (*S*_*i*_) were comprised of new births (*B*_*i*_) without immunity (proportion 1-*p*) and individuals with decreased immunity. These people could be infected by other infectious individuals with a force of infection *λ*_*i*_, modulated by seasonal force and heterogeneity of contacts patterns^14^. After infection, they progress into a latent stage (*E*_*i*_) of duration 1/σ, then enter the infectious stage (*I*_*i*_) of duration 1/*γ*. Recovered individuals (*R*_*i*_) are considered to have their immunity decreased at a rate of *δ*_*i*_. Natural mortalities (*μ*_*i*_) were imposed in all sectors. The supplementary information file describes the mathematical details, whereas the parameters details are summarized in Table 1.

**Table 1:** Summary of Parameters Used in the Models.

Parameter	Definition / explanation	Value(s)	Notes / Reference
	Population size	57,788,688 in 2013	^13^
*N*_*i*_	Population size of age group *i*-th	0.71%, 0.92%, 0.57%, 4.89%, 26.1%, 23.4%, and 43.4% of the population size for *i* = 1, …, 7 respectively	
*B*_*i*_	Number of new births (only for *i* = 1); *B*_*i*_ = 0 for *i*≠1	1.10%, 1.19%, and 1.07% of the population size per year respectively from 2013 to 2015. Assuming 1% increase on the birth rates every year after 2015.	^13^ 1% increase is an estimate over the past five years
*μ*_*i*_	Natural mortality rate for age group *i*-th	0.705%, 0.347%, 0.099%, 0.050%, 0.032%, 0.066%, and 0.833% per year for *i* = 1, …, 7 respectively	^13^
*S*_*i*_(0)	Initial susceptibility proportion for age group *i*-th	Estimates by MCMC	
*λ*_*i*_	Force of infection for age group *i*-th	—	
*β*_*ij*_	Disease transmission rate from individuals in age group *j*-th to *i*-th	—	
*ϕ*	Infectivity rate	Estimates by MCMC	
*δ*_*i*_	Waning rate of vaccine-derived immunity (only for *i* = 5, 6, and 7); *δ*_*i*_ = 0 for *i*≠1	1.5% per year	^16, 32, 33^
1*/σ*	Length of latent period	9 days	^16, 34, 35^
*1/γ*	Length of infectious period	7 days	^34– 36^
*p*	Average proportion of new births with maternal protection for *i* = 1	73.3%	^7^ An estimate based on the serological surveys conducted in 2010 and 2011
*ω*	Seasonal forcing parameter	Estimates by MCMC assuming a flat prior U(0.01, 0.8)	
*ν* ^1^	Vaccine efficacy of MCV1	0.85	^16, 37, 38^
*c* ^1^	Coverage level o of MCV1	95%	An average value reported in the Electronic Surveillance System
*ν* ^2^	Vaccine efficacy of MCV2	0.95	^16, 37, 38^
*c* ^2^	Coverage level o of MCV2	95%	An average value reported in the Electronic Surveillance System
*ν* ^SIA^	Vaccine efficacy of a SIA	0.95	^24^
*c* ^SIA^	Coverage level of a SIA	Test at 20%, 50% and 90%	

### Routine immunization

In China, two doses of MCV are administrated sequentially when children are 8 and 18–24 months of ages. The vaccine efficacies of the two doses are denoted as *ν*^1^ and *ν*^2^, with coverage levels of *c*^1^ and *c*^2^ in the population, respectively. Vaccinated subjects advance to their corresponding recovered compartments with rates *ν*^1^*c*^1^ and *ν*^2^*c*^2^ for MCV1 and MCV2, respectively.

### Model calibration for baseline scenario

The baseline scenario generated by the disease transmission model was calibrated that projects measles epidemics out to the year 2035. Model parameters were estimated using the Markov Chain Monte Carlo method in a Bayesian inferential framework. A likelihood function was formed by assuming a Poisson distribution for measles incidences. Flat prior distributions were assumed for all parameters in the Markov Chain Monte Carlo estimation (Table 1). A random walk Metropolis algorithm was used to obtain the posterior distributions. A total of 10,000 iterations were used as the burn-in period, and 100,000 subsequent iterations were used to draw the posterior estimates. The supplementary information file describes the estimation details. The overall model fitness was assessed using the correlation coefficient (*r*), mean absolute error, and root mean square error.

### Testing of SIA scenarios

A number of SIA scenarios were tested on the baseline scenario of the calibrated model. The primary assessment evaluated the effectiveness of SIAs targeting adults aged >29 years. The efficacies of SIAs targeting school children and teenagers (aged 6–19 years), young adults (20–29 years old), and all three aforementioned age groups were also investigated. As World Health Organization recommended a 2–4-year cycle for SIA implementation^15^, the efficacies of 2- and 4-year cycles were compared. When the model was given *ν*^SIA^ as the vaccine efficacy and *c*^SIA^ as the SIA coverage level, some proportion (*ν*^SIA^*c*^SIA^) of SIA-targeted individuals were designated as recovered. Coverage levels were primarily tested and interpreted at 90% (historical assumption), 50%, and 20% (worst-case scenario) for the targeted populations. The averted incidences (i.e. annual incidence reduction between baseline scenario (no SIA) and SIA scenarios) by years were also assessed at different coverage levels. The SIAs were assumed to start in 2016, with each SIA lasting one month based on previous CDC experience. The impact of late SIAs were also assessed by setting the start year as 2020.

### Parameter uncertainty

A multivariate sensitivity analysis was conducted to assess the influence of major parameter uncertainties on the results. Fitted parameters were assumed to follow their posterior distributions. Plausible fixed-parameter distributions were assumed for the given value ranges^16^. The latency period was assumed to follow a Gamma distribution with a standard deviation of 3 days, whereas uniform distributions (minimum, maximum) were assumed for the waning immunity rate (0.5%, 5%), maternal immunity (70%, 77%), first (0.8, 0.9) and the second (0.9, 0.99) Measles-Mumps-and-Rubella (MMR) vaccine efficacies and their corresponding coverages (0.85, 0.99). Variations in the results were summarized by the interquartile ranges (IQRs) of the annual incidence rates in 2020, 2024, 2028, and 2032 across 1,000 realizations of each scenario.

The statistical analysis was programmed using R software version 3.0.3^17^. These computer programs can be obtained upon request. The surveillance data in this study are available from Hubei CDC and are not publicly available.

## Results

### Model fit and baseline scenario

Figure 1 shows the observed data and model fitted results. The baseline model fit appeared alongside trends among age-stratified cases, as the *r*, mean absolute error, and root mean square error were 0.75, 6.1, and 9.8 respectively. Parameter estimates are summarized in Table 2.

**Table 2:** Estimates and 95% CI in the MCMC estimation.

Parameter	Estimate (95% CI)
Infectivity parameter (*ϕ*)	0.184 (0.182, 0.186)
Seasonal forcing parameter (*ω*)	0.224 (0.208, 0.240)
Initial susceptibility of individuals aged <8 months (*S*_*1*_(0))	42.2% (40.3%, 43.9%)
Initial susceptibility of individuals aged 8 to 17 months (*S*_2_(0))	0.97% (0.04%, 3.31%)
Initial susceptibility of individuals aged 18 to 23 months (*S*_3_(0))	8.9% (5.3%, 12.0%)
Initial susceptibility of individuals aged 2 to 5 years (*S*_4_(0))	2.1% (1.5%, 2.9%)
Initial susceptibility of individuals aged 6 to 19 years (*S*_5_(0))	0.0043% (0.0002%, 0.0223%)
Initial susceptibility of individuals aged 20 to 29 years (*S*_6_(0))	0.0072% (0.0003%, 0.0376%)
Initial susceptibility of individuals aged >29 years (*S*_7_(0))	0.0090% (0.0003%, 0.0468%)

**Figure 3:**
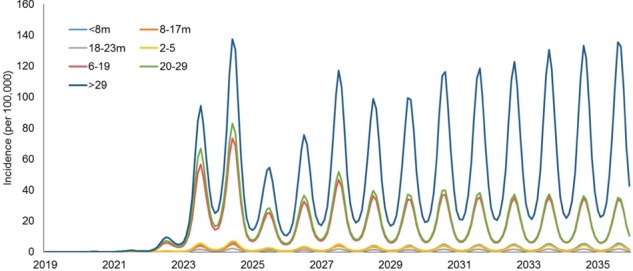
Baseline projections of weekly measles incidences per 100,000 individuals, stratified by age groups.

Figure 3 depicts the baseline measles incidence projections of different age groups. These results indicate that two-dose immunization strategies are insufficient for controlling measles epidemics in the community due to the susceptible percentage growth each year. Epidemic peaks were greater than 10/100,000 for some age groups after 2022. The adult group (>29 years of age) was the primary source driving seasonal epidemics, which could generate >100/100,000 incidence per week at peak levels after 2024. Young adults (20–29 years) and school children and teenagers (6–19 years) were the secondary sources of seasonal epidemics, generating approximately 40/100,000 incidences per week in the Hubei province. Conversely, the weekly measles infections from the age groups of children maintained low levels (<10/100,000) throughout the study period.

### SIA Scenarios

**Figure 4:**
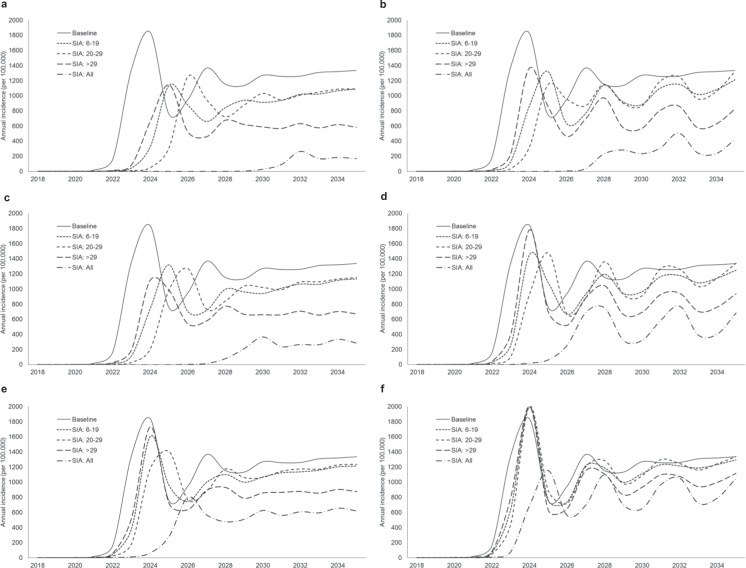
Annual incidence rates for different SIA coverage levels applied to different age groups. SIA coverage levels: 90% (**a**,**b**), 50% (**c**,**d**), and 20% (**e**,**f**). Left (**a**,**c**,**e**) and right (**b**,**d**,**f**) panels respectively refers to 2- and 4-year SIA cycles.

Simulations tested several SIA scenarios targeting different age groups at 2- or 4-year intervals with varying coverage levels (Fig. 4). Among the three targeted groups, administrating SIAs to adults >29 years old every 2 years resulted in the greatest annual incidence rate decrease, maintaining approximately 600/100,000 cases/year after 2025 assuming a 90% coverage level. Although SIAs targeting individuals 20–29 years old failed to substantially reduce rates in the long run, they were able to defer the major epidemic peak from year 2023 to 2026. Given the high coverage levels, incidences could be kept below 10/100,000 until 2030 if 2-year cycle SIAs were provided to all targeted groups (Fig. 4a).

If the 2-year SIA coverage levels dropped to 50%, most SIA scenarios demonstrated little change in incidence reduction compared to scenarios with 90% coverage (Fig. 4c); however, seasonal epidemics grew faster, peaking in 2030 even with SIAs targeting on all studied age groups (age ≥6 years). In the worst-case scenario (20% coverage), SIAs for young adults, school children, and teenagers provided only a slight reduction (averaging ~50/100,000 cases/year) as compared to the baseline scenario. The SIAs targeting individuals 20–29 years old, or for all targeted groups, reduced annual incidence rates by approximately 30% and 50%, respectively, after year 2025 (Fig. 4e).

Generally, 2-year cycle SIAs smoothed the strong seasonal annual incidence patterns relative to 4-year SIAs, even though their average annual incidence reductions were similar (Fig. 4). Like 2-year cycle of SIAs, the impacts of 90% and 50% coverages were similar for SIAs administered to single age groups every 4 years. However, measles incidences increased faster in these scenarios relative to the 2-year cycle SIAs, leveling off at >200/100,000 after year 2028, when SIAs were provided for all targeted groups (Fig. 4b). Incidence rates reached approximately 800/100,000 in 2027 assuming 50% coverage levels (Fig. 4d). In the worst-case scenario with only 20% SIA coverage, SIAs targeting single age groups were unable to observably reduce the incidence levels; however, SIAs targeting all groups still delayed measles epidemic peaks for approximately 1 year and kept the annual incidence below 1% at most times throughout the investigated timespan.

**Figure 5:**
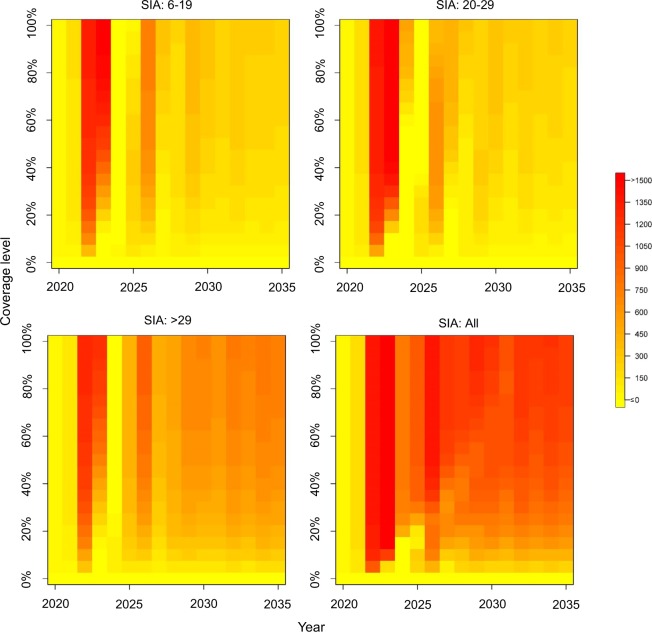
Heat-maps of annual averted incidence (per 100,000 individuals) by years at different coverage levels for SIAs with 2-year cycle.

Figure 5 presents the annual averted incidence by years at different coverage levels for SIAs with 2-year cycle. As SIAs prolonged the inter-epidemic period, the SIAs achieve its greatest averted incidence in the early years. In general, if the coverage of SIAs could be kept above 60%, the averted incidences by years were quite similar at different coverage levels. Although the population size of adults >29 years old was greater than that of the other two groups, varying the coverage level of SIAs to adults did not show higher variation in the averted incidence. In 2030, the averted incidence was around 605/100,000 for SIAs with 50% coverage on adults >29 years old, whereas it was 697/100,000 for SIAs with 90% coverage on adults >29 years old. Nevertheless, the effect of varying coverage levels was more apparent when SIAs were provided for all targeted groups.

The impact of late-application SIAs, administered in year 2020, were also investigated (see Figure S1 in the supplementary information file). As expected, the measles epidemics grew faster than when SIAs started in 2016, although most scenarios showed little incidence reduction. The peak annual measles incidence times in these SIAs scenarios aligned with those seen in baseline scenarios for the worst-case coverage setting.

### Multivariate sensitivity analysis

The results of the multivariate sensitivity analysis from the influence of parameter uncertainties are shown in Figure S2 of the supplementary information file. The major conclusions of this study were generally maintained as the incidence reduction trend remained unchanged when parameter values were altered in a multivariate manner. Larger variations in annual incidence were observed for earlier years, especially in worst-case coverage scenarios, with the IQR of the baseline scenario for year 2020 ranging between 82–2,600/100,000 cases. These uncertainties tended to be greatly reduced in later years. When 2-year cycle SIAs were provided to all targeted groups, the annual incidence IQRs ranged between 10–300/100,000 in 2028 and 2032.

## Discussion

In recent decades, the strategy of supplementing routine immunizations with province-level SIAs has achieved a great success in reducing measles cases in China. This study primarily evaluated the effectiveness of SIAs targeting adults through a mathematical model, calibrated using surveillance data from Hubei (a high-risk province in central China), studying the herd immunity effect. These results showed that future measles epidemics in the community were primarily driven by adults >29 years in age. Compared with SIAs targeting other age groups, adult-targeting (>29 years) SIAs were the most effective in terms of reducing annual incidence rates. In most scenarios, SIAs targeting this group could reduce incidences by half in the long run. Nevertheless, to maintain very low annual measles infection levels in China, SIAs should be provided every 2 years to individuals ≥6 years of age, and each SIA should maintained at least 50% coverage. These findings may be used to advise officials in the prioritization of mass vaccination campaigns for appropriate populations.

This study demonstrated the effectiveness of SIAs targeting adults, primarily due to the increasing infection age in China documented by many prior investigations^6,7,12,18,19^. Ma *et al*. indicated infected adults ≥20 years old accounted for 43% of all incidences in 2014^6^. Recently, in north-eastern and western China, adults were found to be a major source of infections during the measles outbreaks^18–20^. The increasing proportion of adult measles cases was due to the increase of susceptibles in this group, which was a collective result of: i. missed vaccinations in the past decades^18^, and ii. the decreased chance of acquiring immunity through natural infections, which was further due to the decreased birth rate and improved immunization among newborns^12^. China introduced liquid measles vaccines in 1965 and implemented the single-dose routine immunization schedule using liquid vaccines in 1978 and the two-dose schedule using lyophilized vaccine in 1986^6^. Yet the vaccination rate was low during the 1960s to 1980s, especially among sub-urban population^18^, and the estimated MCV1 coverage was around 80% in 2000^6^. Thus, cohorts that were born after 1965 and before 1996, who were around 20 to 50 years old in 2015, had a low vaccination coverage rate yet were not covered by the SIAs (implemented between 2004–2010). The 20–40 years old, whom were at their child-bearing age, were the most susceptible^21^. Many might have missed the vaccinations (both routine immunizations and SIAs) but remained susceptible in their adulthood due to the reduced chance of natural infection as vaccination rates improved among the children. Serological surveys conducted in the Hubei^7^, Tianjin^9^, and Jiangsu^21^ provinces also revealed low seropositivity rates among adults. In China, adults born before 1995, >25 years old in 2020, did not receive national SIAs (implemented between 2004–2010) and may thus be infected due to an absence of acquired immunity from previous infections or missed routine immunizations. As a result, adults 20–40 years old (of child-bearing age) are likely the most vulnerable group^22^. The large population in this age range, alongside their often-unknown immunization histories, poses a significant obstacle to mass vaccination, prompting the need for a more specific immunization program^21^.

To this end, this study demonstrated that adult-targeting SIAs would become most effective in the long run. Many SIAs were extended toward adult groups in Europe (<45 years old), some of which reduced case prevalence to <1/1,000,000^23^. The current situation in China resembles that in Europe from past decades – despite the high coverages of routine MCV programs, measles incidence remained high until the adult immunity gap was addressed by SIAs. Nevertheless, the feasibility of adult-targeting SIAs remains under question in China. First, a voluntary campaign may not attract enough subjects to achieve coverage sufficient for herd immunity, even though this study demonstrated moderate coverages are still able to obtain a desirable incidence reduction. Second, non-selective SIAs can require many repeated vaccinations, result in high unnecessary costs^20,23^. The increasing risk of adverse events and large re-vaccination campaigns would increase public concern and likely have a high media profile. Third, high migration rates in urban districts pose a logistic difficulty for adult-targeted SIAs^18^. Therefore, individual revaccinations must include well-planned identification and community-wide monitoring for adult infections and vaccine-related adverse events.

This study showed the seasonal oscillations of annual incidence differed between 2- and 4-year SIA cycles in the setting of the Hubei province, likely due to accumulation of susceptible adults over 4-year inter-SIA periods. These results aligned with those of Verguet *et al*.^24^ showing the impacts of SIAs were highly depended on population demography and 4-year SIA cycles could potentially result in several measles resurgences of epidemic proportions. These results may have differed from those described in some modelling studies where different interventions were evaluated simultaneously, especially those including case-triggered measures;^25^ however, we demonstrated that regular SIAs targeting individuals ≥6 years old could control measles epidemic outbreaks for a long period of time, and thereby provide a better option than case-triggered campaigns which are likely to have delayed responses to laboratory-based triggers. Furthermore, outbreak-triggered campaigns may not be able to avert significant numbers of cases in China, which has already implemented high coverage routine immunizations^25^, as opposed to middle- and low-income countries where outbreak-triggered campaigns have traditionally been supported^26^. SIAs may thus be more preferred in China, a country where measles has not yet been eliminated. A cost-effectiveness analysis accounting for resources and logistic constraints should thus be conducted to evaluate different combinations of control strategies, especially in areas where measles has not yet been eliminated.

Mathematical models evaluating interventions require biological plausibility, and thus must account for the abstraction and generalization of realism. This study thus encountered several major limitations. First, the simulation outcome was calibrated using CDC data, though incidences were likely underreported. Previous studies have indicated gaps exist between official report coverage and survey-based assessments^18,20^. The baseline simulations might thus have contained underestimated; however, sensitivity analyses were performed to assess the worse scenarios. Second, variations in measles transmissions could be due to many alternative factors including stochasticity, spatial structure^27,28^, dose effect dependences, and social heterogeneity^16^. The current transmission model was a relatively simple method of addressing these current questions and did not modulate for the abovementioned setting factors. Unfortunately, more data points will be required to construct a better model for answering these research questions in detail. Third, although these results demonstrated the children group was not a major driving force of measles epidemics under the assumption of a 1% birth rate (the average from the past 5 years), birth rate remains a key determinant in major epidemic sizes, and variations in it may affect simulation outcomes^27^. In 1979, China adopted the one-child policy (i.e. family planning for one-child-per-couple) and has recorded a continuously decreasing birth rate, resulting in a declining overall prevalence of measles prevalence and shifted age distribution, with adult cases increasingly predominating. The increased mean of age infection was, conversely, found to be a natural consequence of the decreasing overall prevalence thanks to the vaccinations given in the midst of China’s demographic transition, rather than being solely due to waning immunity and SIA efficacy^12^. In late-2015, however, the one-child policy was replaced by a universal two-child policy and the fertility rate was expected to increase from the 2016 levels of 1.24 births in urban areas and 2.01 in rural areas to 1.67 and 2.15, respectively, by 2026^29^. Measles dynamics depend largely on demographic characteristics, and the increasing birth rate and altered age distribution were assumed to increase transmission rates and affect outbreak patterns and cycles, prompting irregular disease dynamics when birth rates increased rapidly^27^. Liu *et al*., however, recently demonstrated the two-child policy generated a negligible difference in the annual influenza attack rate relative to the one-child policy, probably due to the decreased household size and proportion of children in the community^30^. Relative to influenza, measles is a pediatric infectious disease whose transmission is primarily driven by children and occurs in health institutions and schools. Such heterogeneities, in addition to the diseases characteristics, induce different impacts on incidence rates. Future studies must be conducted to incorporate the structural details of the contact network for a further exploration. Lastly, the modelling results were used to inform the policy from the effectiveness assessment of SIAs and we do not intent to optimize the population coverage and schedule of vaccination. More extensions for optimizing the intervention on lowering the transmissibility or even on eliminating measles warrant an investigation in the future.

Mathematical modelling studies help officials to prioritize mitigation strategies. In conclusion, adult-targeted (>29 years) two-year SIA cycles produced the greatest annual incidence rate decrease, reducing incidences by half over a long timespan with 90% coverage levels. Incidences could remain below 10/100,000 until 2030 if SIAs were provided to individuals ≥6 years old with at least 50% coverage. Hence, the findings suggest that public health officials in China should remain alert to adult-to-adult measles transmissions. Although officials have reported approximately 90% coverage for past SIAs, this does not necessarily reflect a reduction of susceptible individuals, primarily because a certain proportion of individuals may retain immunity from previous immunizations or natural exposure^31^. Future adult-targeting SIAs should be provided at shorter intervals with enhanced surveillance, particularly considering older adults with decreased immunity.

## Electronic supplementary material

Supplementary Information File
