# Supplementary material for: Targeting Adults for Supplementary Immunization Activities of Measles Control in Central China: A Mathematical Modelling Study
Source: Sci Rep. 2018 Oct 31;8:16124. doi: 10.1038/s41598-018-34461-0 (PMC6208397; doi:10.1038/s41598-018-34461-0)

Targeting Adults for Supplementary Immunization Activities of Measles Control in Central China: A Mathematical Modelling Study

Authors and affiliations:

Ka Chun Chong [a,b,1], Chi Zhang [c,1], Katherine Min Jia [a], Benny Chung Ying Zee [a,b], Tongyong Luo [a], Lei Wang[c], Greta Chun Huen Tam [a], Riyang Sun [a,b], Maggie Haitian Wang [a,b*], Xuhua Guan [c*]

a JC School of Public Health and Primary Care, The Chinese University of Hong Kong, Hong Kong, China

b Clinical Trials and Biostatistics Laboratory, Shenzhen Research Institute, The Chinese University of Hong Kong, China

c Hubei Provincial Center for Disease Control and Prevention, Hubei, China

Supplementary Information File – Mathematical details for the disease transmission models and supplementary figure S1-S2

Disease transmission model

An age-structured compartmental model was used to study the transmission dynamics and the impacts of SIA interventions. Susceptible individuals in age group *i*-th (S_i) comprised new births (B_i) that with no immunity (proportion 1-*p*) and individuals having immunity waned. These people could be infected by other infectious individuals with a force of infection λ_i, and then progress into a latent stage (E_i) of duration 1/σ. Following the latent period, they are infectious (I_i) of duration 1/γ. Recovered individuals (R_i) are considered to have their immunity decreased at a rate of δ_i. Natural mortality of each age group (μ_i) are imposed to all compartments. The dynamic equations are

$$\begin{aligned}\frac{dS_i}{dt} &= (1-p)B_i - (\mu_i + \lambda_i)S_i + \delta_i R_i \\ \frac{dE_i}{dt} &= \lambda_i S_i - (\sigma + \mu_i)E_i \\ \frac{dI_i}{dt} &= \sigma E_i - (\gamma + \mu_i)I_i \\ \frac{dR_i}{dt} &= pB_i + \gamma I_i - (\mu_i + \delta_i)R_i\end{aligned}$$

The transmission of individuals among age groups is

$$\begin{aligned}\mathrm{S}_{i+1} &\rightarrow \mathrm{S}_{i+1} + \mathrm{S}_i\left(\frac{\Delta t}{\Delta T_i}\right) - \mathrm{S}_{i+1}\left(\frac{\Delta t}{\Delta T_{i+1}}\right) \\ \mathrm{E}_{i+1} &\rightarrow \mathrm{E}_{i+1} + \mathrm{E}_i\left(\frac{\Delta t}{\Delta T_i}\right) - \mathrm{E}_{i+1}\left(\frac{\Delta t}{\Delta T_{i+1}}\right) \\ \mathrm{I}_{i+1} &\rightarrow \mathrm{I}_{i+1} + \mathrm{I}_i\left(\frac{\Delta t}{\Delta T_i}\right) - \mathrm{I}_{i+1}\left(\frac{\Delta t}{\Delta T_{i+1}}\right) \\ \mathrm{R}_{i+1} &\rightarrow \mathrm{R}_{i+1} + \mathrm{R}_i\left(\frac{\Delta t}{\Delta T_i}\right) - \mathrm{R}_{i+1}\left(\frac{\Delta t}{\Delta T_{i+1}}\right)\end{aligned}$$

where ΔT_i is the time width in terms of time step Δt.

Force of infection

In China, measles infection had a strong seasonal variation in which the peaks usually occurred in April every year. Seasonal forcing is commonly incorporated into disease transmission models through the transmission rate, either using a square wave term or a sinusoidal term to allow for a time-dependent of infection pattern [3, 4]. The adaption of the seasonal forcing helps producing stable oscillations affected by the interaction between transmissions and variation from some factors such as school holiday [5, 6] and climate [7, 8], which could not be comprehensively modulated in the model.

The force of infection (λ_i) of age group *i*-th was adjusted with the seasonal forcing function:

$$\omega(t) = 1 + \omega \sin\left(\frac{2\pi t}{T}\right)$$

where ω is the seasonal forcing parameter and T equals to one year. The ω was estimated through model fitting and the seasonal forcing function corresponds to be maximum in March to May and to be minimum in September to November.

Suppose β_{ij} is the disease transmission rate from individuals in age group *j*-th to *i*-th, then

$$\lambda_i = \omega(t)\sum_{j=1}^{7}\frac{\beta_{ij} I_j}{N_j}$$

given N_j is the population size of age group *j*-th. Because of different contact patterns, a who acquires infection from whom (WAIFW) matrix was used. WAIFW matrix is commonly employed in a disease transmission model to take account of the heterogeneity of transmission pattern when an infectious disease is highly age-dependent. An early study has showed that majority of children should be vaccinated well before the age of first attendance at school for a measles eradication by using an age-structured disease transmission model [9]. The use of WAIFW matrix has been further extended to adapt

different contact patterns [10]. By using a recent published social contact data [1], the WAIFW matrix was constructed by the following:

$$\beta^{M} = \begin{pmatrix} \beta_{11} & \beta_{12} & \beta_{13} & \beta_{14} & \beta_{15} & \beta_{16} & \beta_{17} \\ \beta_{21} & \beta_{22} & \beta_{23} & \beta_{24} & \beta_{25} & \beta_{26} & \beta_{27} \\ \beta_{31} & \beta_{32} & \beta_{33} & \beta_{34} & \beta_{35} & \beta_{36} & \beta_{37} \\ \beta_{41} & \beta_{42} & \beta_{43} & \beta_{44} & \beta_{45} & \beta_{46} & \beta_{47} \\ \beta_{51} & \beta_{52} & \beta_{53} & \beta_{54} & \beta_{55} & \beta_{56} & \beta_{57} \\ \beta_{61} & \beta_{62} & \beta_{63} & \beta_{64} & \beta_{65} & \beta_{66} & \beta_{67} \\ \beta_{71} & \beta_{72} & \beta_{73} & \beta_{74} & \beta_{75} & \beta_{76} & \beta_{77} \end{pmatrix} = \phi \begin{pmatrix} c_{11} & c_{12} & c_{13} & c_{14} & c_{15} & c_{16} & c_{17} \\ c_{21} & c_{22} & c_{23} & c_{24} & c_{25} & c_{26} & c_{27} \\ c_{31} & c_{32} & c_{33} & c_{34} & c_{35} & c_{36} & c_{37} \\ c_{41} & c_{42} & c_{43} & c_{44} & c_{45} & c_{46} & c_{47} \\ c_{51} & c_{52} & c_{53} & c_{54} & c_{55} & c_{56} & c_{57} \\ c_{61} & c_{62} & c_{63} & c_{64} & c_{65} & c_{66} & c_{67} \\ c_{71} & c_{72} & c_{73} & c_{74} & c_{75} & c_{76} & c_{77} \end{pmatrix}$$

where β_{ij} is the disease transmission rate from individuals in age group *j*-th to individuals in age group *i*-th per unit time. β_{ij} depends on both the frequency of contact with others and the proportion of those contacts that are effective (i.e. sufficient to infect an individual when the contact occur between an infectious and a susceptible individual. Hence, β_{ij} can be formulated as a product between c_{ij}, average number of contacts from individuals in age group *j*-th to *i*-th and ϕ, the infectivity rate. The values of c_{ij} were interpolated from the data published by Prem et al. [1].

Equations for routine immunization

In China, MCV1 and MCV2 are recommended with the first and second doses respectively administrated at 8 and 18 to 24 months of ages. Suppose v^1 and v^2 are vaccine efficacies and c^1 and c^2 are coverage levels in the population respectively, the equation for susceptible compartment of age group 8 to 17 months is

$$\frac{dS_2}{dt} = -\left(\mu + \lambda_2 + v^1 c^1\right) S_2$$

and of age group 18 to 23 months is

$$\frac{dS_3}{dt} = -\left(\mu + \lambda_3 + v^2 c^2\right) S_3$$

Those vaccinated subjects will advance to their corresponding recovered compartments.

Model calibration

The model parameters were estimated using Markov Chain Monte Carlo (MCMC) method in a Bayesian inferential framework. The observed measles incidence data ($x_i(t)$) were fitted against the model generated incidence i.e. $\eta_i(t) = S_i(t)\lambda_i(t)$ for each of day *t*. A likelihood function was formed by assuming Poisson-distributed measles incidence:

$$L(\boldsymbol{\beta}, \omega) = \prod_i \prod_{t=1} \frac{e^{-\eta_i(t)} \eta_i(t)^{x_i(t)}}{x_i(t)!}$$

In the MCMC estimation, flat prior distributions were assumed for all parameters (Table 1). A random walk Metropolis algorithm was used to obtain the posterior distributions. Totally 10000 iterations were used as the burn-in period and the subsequent 100 000 iterations were used to draw the posterior estimates. Step sizes were selected to obtain acceptance proportions between 20% and 40%. The convergence of the mixing of the MCMC chains was diagnosed with time-series trace plots and autocorrelation functions. The medians and 95% credible intervals (CIs) of the posterior distributions were used to summarize the estimates.

SIA scenarios

A number of SIA scenarios were tested on the baseline scenario of the calibrated model. The assessment of the simulation is to evaluated the effectiveness of SIAs targeting different age groups: school children and teenagers (aged 6–19 years), young adults (20–29 years old), adults aged >29 years, and all of them. When the model was given v^{SIA} as the vaccine efficacy and c^{SIA} as the SIA coverage level, a proportion ($v^{\mathrm{SIA}}c^{\mathrm{SIA}}$) of individuals in an age group was targeted by the periodical SIAs and they enter the recovered stage:

$$\frac{dS_i}{dt} = (1 - p)B_i - \left(\mu_i + \lambda_i + v^{SIA} c^{SIA}\right)S_i + \delta_i R_i$$
$$\ldots$$
$$\frac{dR_i}{dt} = pB_i + \gamma I_i - (\mu_i + \delta_i)R_i + v^{SIA} c^{SIA} S_i$$

We tested 2-year and 4-year cycles of SIA per World Health Organization recommendation [2]. Coverage levels (c^{SIA}) were tested at 90% (historical assumption), 50%, and 20% (worst-case scenario) for the targeted populations. The SIAs were assumed to start in 2016, with each SIA lasting one month based on previous CDC experience. The impact of late SIAs were also assessed by setting the start year as 2020.

Reference:

1. Prem K, Cook AR, Jit M. Projecting social contact matrices in 152 countries using contact surveys and demographic data. PLoS computational biology. 2017 Sep 12;13(9):e1005697
2. World Health Organization. Measles vaccines: WHO position paper. Wkly Epidemiol Rec. 84, 349-360 (2009).
3. Roberts MG, Tobias MI. Predicting and preventing measles epidemics in New Zealand: application of a mathematical model. Epidemiology & Infection. 2000;124(2):279-87.
4. Ferguson NM, Nokes DJ, Anderson RM. Dynamical complexity in age-structured models of the transmission of the measles virus: epidemiological implications at high levels of vaccine uptake. Mathematical biosciences. 1996;138(2):101-30.
5. Fine PE, Clarkson JA. Measles in England and Wales—I: an analysis of factors underlying seasonal patterns. International journal of epidemiology. 1982;11(1):5-

14.

6. Finkenstädt BF, Grenfell BT. Time series modelling of childhood diseases: a dynamical systems approach. Journal of the Royal Statistical Society: Series C (Applied Statistics). 2000;49(2):187-205.
7. Yang Q, Fu C, Wang N, Dong Z, Hu W, Wang M. The effects of weather conditions on measles incidence in Guangzhou, Southern China. Human vaccines & immunotherapeutics. 2014 Apr 28;10(4):1104-10.
8. De Jong JG. The survival of measles virus in air, in relation to the epidemiology of measles. Archives of Virology. 1965 Feb 1;16(1):97-102.
9. Anderson RM, May RM. Age-related changes in the rate of disease transmission: implications for the design of vaccination programmes. Epidemiology & Infection. 1985;94(3):365-436.
10. Mossong J, Hens N, Jit M, Beutels P, Auranen K, Mikolajczyk R, Massari M, Salmaso S, Tomba GS, Wallinga J, Heijne J. Social contacts and mixing patterns relevant to the spread of infectious diseases. PLoS medicine. 2008 Mar 25;5(3):e74.

Supplementary Figures:

Figure S1. Annual incidence rates when SIAs were applied lately in 2020 to different age groups with 90% (A and B), 50% (C and D), and 20% (E and F) coverage levels. Left (A, C, and E) and right (B, D, and F) panels respectively refers to 2-year and 4-year cycle of SIAs.

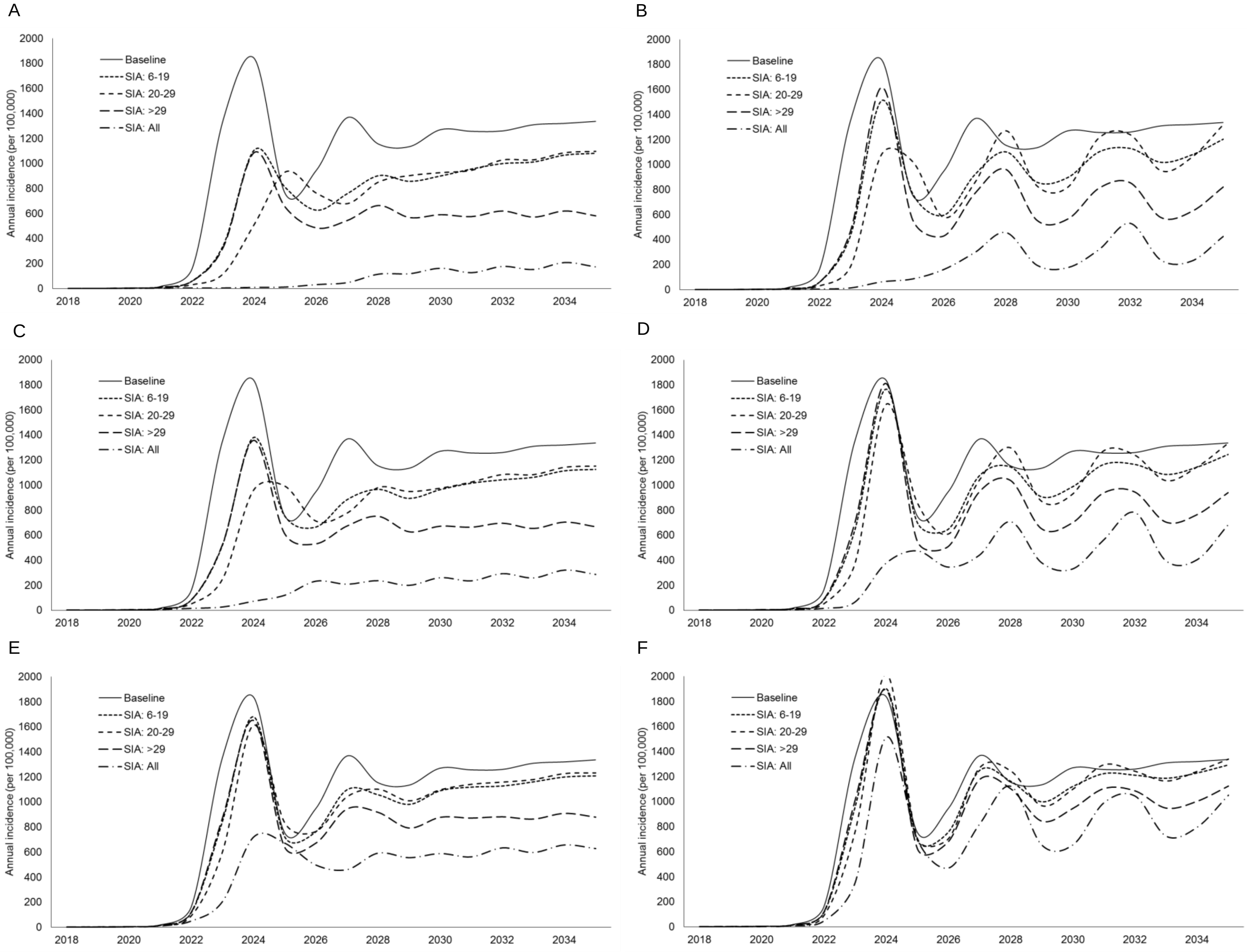

Figure S2. Interquartile ranges of the annual incidence rates at year 2020, 2024, 2028, and 2032 under different scenarios of2-year cycle of SIAs in the sensitivity analysis. 90%, 50%, and 20% coverage levels are presented at upper, middle and lower panels respectively.

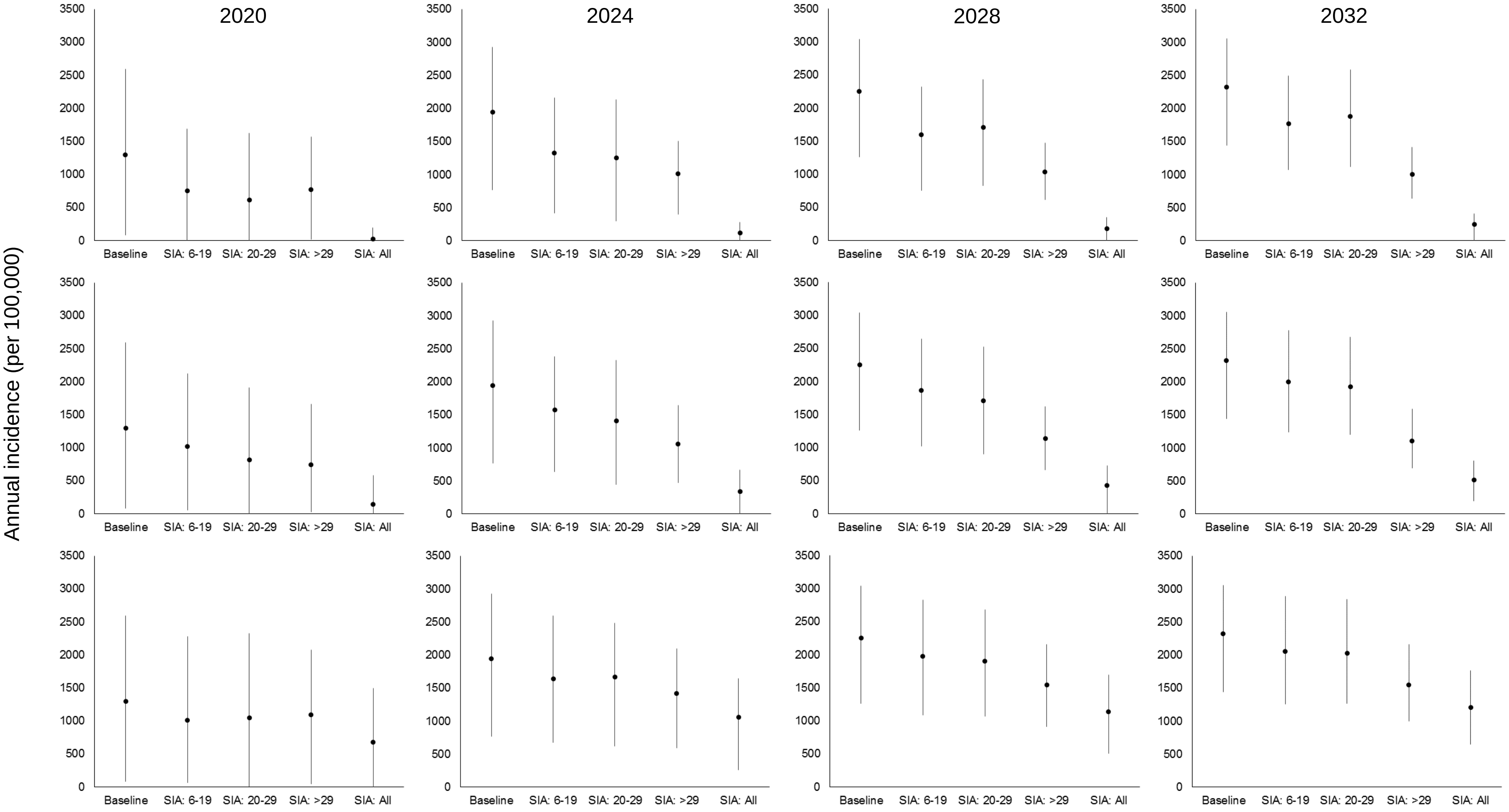

Supplement: Supplementary file 1 — Supplementary Information File [file 41598_2018_34461_MOESM1_ESM.pdf]
